# Melting pot of tick-borne zoonoses: the European hedgehog contributes to the maintenance of various tick-borne diseases in natural cycles urban and suburban areas

**DOI:** 10.1186/s13071-017-2065-0

**Published:** 2017-03-07

**Authors:** Setareh Jahfari, Sanne C. Ruyts, Ewa Frazer-Mendelewska, Ryanne Jaarsma, Kris Verheyen, Hein Sprong

**Affiliations:** 1 Centre for Infectious Disease Control, National Institute of Public Health and Environment (RIVM), Antonie van Leeuwenhoeklaan 9, 3720 BA Bilthoven, The Netherlands; 2 Forest and Nature Laboratory, Department of Forest and Water Management, Ghent University, Geraardsbergsesteenweg 267, 9090 Melle-Gontrode, Belgium

**Keywords:** *Anaplasma phagocytophilum*, *Borrelia burgdorferi* (*sensu lato*), *Borrelia miyamotoi*, “*Candidatus* Neoehrlichia mikurensis”, *Rickettsia helvetica*, European hedgehog, *Erinaceus europaeus*, Lyme borreliosis, *Ixodes hexagonus*, *Ixodes ricinus*

## Abstract

**Background:**

European hedgehogs (*Erinaceus europaeus*) are urban dwellers and host both *Ixodes ricinus* and *Ixodes hexagonus*. These ticks transmit several zoonotic pathogens like *Borrelia burgdorferi* (*sensu lato*), *Anaplasma phagocytophilum*, *Rickettsia helvetica*, *Borrelia miyamotoi* and “*Candidatus* Neoehrlichia mikurensis”. It is unclear to what extent hedgehogs in (sub) urban areas contribute to the presence of infected ticks in these areas, which subsequently pose a risk for acquiring a tick-borne disease. Therefore, it is important to investigate to what extent hedgehogs contribute to the enzootic cycle of these tick-borne pathogens, and to shed more light at the mechanisms of the transmission cycles involving hedgehogs and both ixodid tick species.

**Methods:**

Engorged ticks from hedgehogs were collected from (sub) urban areas *via* rehabilitating centres in Belgium. Ticks were screened individually for presence of *Borrelia burgdorferi* (*sensu lato*), *Borrelia miyamotoi*, *Anaplasma phagocytophilum, Rickettsia helvetica* and “*Candidatus* Neoehrlichia mikurensis” using PCR-based methods. Infection rates of the different pathogens in ticks were calculated and compared to infection rates in questing ticks.

**Results:**

Both *Ixodes hexagonus* (*n* = 1132) and *Ixodes ricinus* (*n* = 73) of all life stages were found on the 54 investigated hedgehogs. Only a few hedgehogs carried most of the ticks, with 6 of the 54 hedgehogs carrying more than half of all ticks (624/1205). *Borrelia miyamotoi*, *A. phagocytophilum*, *R. helvetica* and *B. burgdorferi* genospecies (*Borrelia afzelii*, *Borrelia bavariensis* and *Borrelia spielmanii*) were detected in both *I. hexagonus* and *I. ricinus. Anaplasma phagocytophilum*, *R. helvetica*, *B. afzelii*, *B. bavariensis* and *B. spielmanii* were found significantly more in engorged ticks in comparison to questing *I. ricinus*.

**Conclusions:**

European hedgehogs seem to contribute to the spread and transmission of tick-borne pathogens in urban areas. The relatively high prevalence of *B. bavariensis*, *B. spielmanii*, *B. afzelii*, *A. phagocytophilum* and *R. helvetica* in engorged ticks suggests that hedgehogs contribute to their enzootic cycles in (sub) urban areas. The extent to which hedgehogs can independently maintain these agents in natural cycles, and the role of other hosts (rodents and birds) remain to be investigated.

**Electronic supplementary material:**

The online version of this article (doi:10.1186/s13071-017-2065-0) contains supplementary material, which is available to authorized users.

## Background

The incidence of tick-borne diseases has increased the last decades and poses important economic and medical consequences [1]. Lyme borreliosis is the most prevalent tick-borne disease in Europe and presents itself under a wide range of clinical manifestations. The most common and earliest manifestation is an expanding rash at the site of the tick bite (erythema migrans), and left untreated it can progress towards more severe disease manifestations. The disseminated infection can affect a patient’s nervous system, joints, lymph nodes, skin, and in rare cases the heart or eyes [2]. The causative agents of Lyme borreliosis are spirochetes belonging to the *Borrelia burgdorferi* (*sensu lato*) (*s.l*.) complex. They are generally transmitted by ticks of the *Ixodes ricinus* complex [3] and are maintained in enzootic cycles by different vertebrate hosts [4–6]. At least five genospecies of *B. burgdorferi* (*s.l*.) complex have been shown to be pathogenic to humans, namely *B. burgdorferi* (*sensu stricto*) (*s.s*.), *B. afzelii, B. garinii*, *B. spielmanii* and *B. bavariensis* [2, 7]. Each of these genospecies is maintained in nature through a distinct enzootic cycle involving ixodid ticks and vertebrates acting as reservoirs [8, 9]. The different *Borrelia* genospecies are generally associated with different clinical manifestations [7]. Current knowledge is that *B. afzelii* is predominantly involved in dermal infections (erythema migrans and acrodermatitis chronica atrophicans) [2] and is adapted to rodents and other small mammals [9, 10]. *Borrelia garinii* is associated with neurological manifestations and birds maintain most of the *B. garinii* strains [9, 11, 12]. *Borrelia bavariensis* is frequently found in rodents [13, 14] and hedgehogs [15] and is also associated with neuroborreliosis in humans [7]. *Borrelia spielmanii* infection in humans is rare, and only found in patients with erythema migrans. Its reservoir hosts are of the family Gliridae [16], but this *Borrelia* genospecies has also been detected in hedgehogs and their ticks [15, 17]. Besides the *B. burgdorferi* (*s.l*.) genospecies, other established pathogens circulate in enzootic cycles including the same ixodid ticks and vertebrate reservoirs, for example *Anaplasma phagocytophilum*, *Rickettsia helvetica*, *Borrelia miyamotoi* and “*Candidatus* Neoehrlichia mikurensis”. These pathogens can cause non-characteristic, viral-like symptoms in humans, and often confused with Lyme borreliosis [18]. Their infections are often self-limiting, but in immunocompromised patients, they can cause severe clinical manifestations [19–22]. In the framework of human health, therefore, it is important to identify the different components of the enzootic cycle of these tick-borne diseases, and to shed more light at the mechanisms of the transmission cycles.

The generalist tick species *I. ricinus* actively quests in the vegetation for hosts to feed on and may readily bite humans, thereby possibly transmitting pathogens. *Ixodes hexagonus* is a host specialist, feeding primarily on hedgehogs. It shows an endophilic behaviour preferring dark, humid places, and is usually found in the nests of its host species [23]. Besides hedgehogs, this tick species has been found to infest other host species as well [24]. Despite their nest dwelling behaviour, it is known to occasionally bite humans and companion animals, even though less frequently than *I. ricinus* does [24, 25]. Both ixodid species can be found on hedgehogs and are competent vectors for transmitting *B. burgdorferi* (*s.l*.) [26–28].

The European hedgehog (*Erinaceus europaeus* Linnaeus, 1758) is a nocturnal insectivorous mammal commonly found throughout Western Europe [29]. They seem to have adjusted to a wide variety of habitats and occur in rural, suburban, and urban areas but generally prefer grassland with sufficient edge habitats. Hedgehogs can reach up to nine times higher densities in urban areas with parks and garden, than in rural areas, with lowest densities in forests and open grassland fields and agricultural land without cover such as shrubs or dead wood [30–32]. Since they are one of the most successful urban adapters, hedgehogs and *I. hexagonus* could contribute to the spread and persistence of pathogens in a (sub) urban habitat *via* secondary enzootic cycles, even when the contact between *I. hexagonus* and humans is low [15, 33].

Only a few studies have been performed on the reservoir competence of the European hedgehog. These studies have shown that these mammals can be infected with different *B. burgdorferi* (*s.l*.) genospecies [15, 17, 23] as well as other tick-borne pathogens, such as *A. phagocytophilum* [34, 35], tick-borne encephalitis virus (TBEV) [28] and *R. helvetica* [36]. The role of the European hedgehog and both ixodid tick species feeding on it in the transmission cycle of many tick-borne pathogens like *B. miyamotoi* and “*Candidatus* Neoehrlichia mikurensis” is not completely illuminated, yet [37].

In this study, we aim to investigate the prevalence of *B. burgdorferi* (*s.l*.) genospecies, *B. miyamotoi*, *A. phagocytophilum*, “*Ca.* Neoehrlichia mikurensis” and *R. helvetica* in the different stages of the *I. hexagonus* and *I. ricinus* tick species sampled from European hedgehogs (*E. europaeus*) in Belgium. Furthermore, we aim to investigate the role of these tick species and that of the hedgehog in the enzootic cycle of the different disease pathogens. By using epidemiological analysis and comparing the infection prevalences of the different pathogens from engorged ticks collected from hedgehogs with questing nymphs from the vegetation, we aim to (i) determine the reservoir status of the European hedgehog for *B. burgdorferi* (*s.l*.) genospecies, *B. miyamotoi*, *A. phagocytophilum*, “*Ca.* Neoehrlichia mikurensis” and *R. helvetica*, and (ii) find indications for the vector competence of *I. hexagonus* for tick-borne pathogens.

## Methods

### Hedgehog and tick sampling

Since European hedgehogs are legally protected in Belgium, the current investigation was carried out on ticks sampled from hedgehogs that were brought to the rehabilitation centres of Herenthout and Heusen-Zolder in the Campine region, Belgium. In general, these hedgehogs were captured in gardens and urban areas by civilians. To grant the hedgehogs an easy and full recovery, removal of all ectoparasites upon arrival at the rehabilitation centre is a standard procedure. For this study, attached ticks of all life stages were collected by the centres’ volunteers in 2014 (both centres) and 2015 (only Herenthout) between the end of April and the end of October. Tick specimens were stored in 70% ethanol at room temperature until further investigation. Ticks were identified to species and life stage [38]. The number of attached ticks (tick burden) was recorded for each hedgehog. Since only hedgehogs that harboured ticks were used in this study, there is no data on the percentage of hedgehogs that were infested by ticks. Age (adult or juvenile) was determined based on weight [15] for all hedgehogs, except two. The questing *I. ricinus* ticks, that were caught by drag-sampling the vegetation in the same region as where the hedgehogs were collected, are part of a previously published study [39].

### Sample preparation and molecular detection of tick-borne pathogens

All ticks were processed individually. Nucleic acids were extracted using the DNeasy Blood & Tissue Kit (Qiagen, Hilden, Germany), according to the manufacturer’s instructions. The extracted DNA was stored at -20 °C until further use. Ticks were tested individually for presence of *B. burgdorferi* (*s.l*.), *B. miyamotoi*, *A. phagocytophilum*, “*Ca.* Neoehrlichia mikurensis” and *R. helvetica* DNA using two separate multiplex real-time PCR assays as described before [39–42], followed by sequencing for species identification. For the identification of *B. burgdorferi* (*s.l*.) genospecies a conventional PCR assay targeting the 5S–23S intergenic region (IGS) was performed (see Additional file 1). *Borrelia burgdorferi* (*s.l*.) genospecies identification was determined by comparison of sequences to isolate in-house molecular databases [43]. As genospecies identification was successful for only 43.4% of the *B. burgdorferi* (*s.l*.) positive sequences, we proportionally assigned these unidentifiable sequences in each life stage per tick species to the different *B. burgdorferi* (*s.l*.) genospecies present in stage, using the proportion of each genospecies detected in that stage as a weighting factor (Hofmeester et al. submitted). We assumed that the probability to successfully identify a genospecies is equal for all *B. burgdorferi* (*s.l*.) genospecies. For confirmation of *B. miyamotoi* conventional PCR targeting *glpQ* gene was done [20]. The *groEL* gene of *A. phagocytophilum* was amplified and sequenced [44] (see Additional file 2). For all conventional PCR’s, both strands of PCR products were sequenced by BaseClear (Leiden, the Netherlands).

### Statistical tests

All statistical tests were performed using R version 3.2.0 (R Core Team, 2016 [45]) and all graphs were made with the package *ggplot2* [46]. To test the differences in distribution of tick species, tick burden and infection prevalence of the different pathogens in ticks on hedgehogs from different age classes, Kruskal-Wallis tests were employed. The number of mixed tick species infestations (both tick species on the same hedgehog) was compared to the number of single species infestations (only *I. ricinus* or *I. hexagonus*) with Pearson’s Chi-square test. With the *prop.test* function, we tested if the pathogens in the ticks occurred more frequently alone or co-existing with a different pathogen in the same tick. Afterwards we compared the infection prevalence of the pathogens in *I. hexagonus* with the prevalence in *I. ricinus.* Finally, to assess the transmission capabilities of the hedgehog for each pathogen, we compared the infection prevalence in the engorged ticks collected from hedgehogs with the infection prevalence in questing *I. ricinus* from the same region [39], and used this as a proxy to evaluate the reservoir status of the hedgehog. To evaluate the reservoir status of a host species, it is best to compare engorged *I. ricinus* larvae with questing *I. ricinus* larvae and nymphs. As the amount of engorged *I. ricinus* larvae in our study is too low to perform these analyses (*n* = 7), we decided to compare the infection prevalence of each pathogen in engorged *I. ricinus* larvae and nymphs collected from hedgehogs with the infection prevalence in host-seeking *I. ricinus* nymphs and adults. This way, we compare ticks that fed once (engorged larvae and questing nymphs) or twice (engorged nymphs and questing adults), and omit ticks that had the chance to feed three times (engorged adults). Engorged adults have a higher chance to be infected than a questing tick anyway, that has never fed more than twice. The difference between the pathogen communities in engorged and questing ticks is thus that the engorged ticks will have certainly fed, at least once, on hedgehogs, while the chance that the questing ticks will have fed on hedgehogs is rather low. Differences between the infection prevalence of the pathogens in engorged and questing ticks may then reflect the importance of the hedgehog in the transmission of pathogens. A higher infection prevalence of a certain pathogen in engorged larvae and nymphs will then suggest that the hedgehog has an important role in the transmission of that pathogen.

## Results

**Fig. 1:**
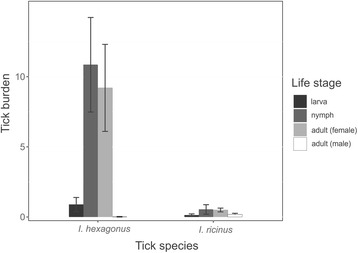
The distribution of the different life stages of *Ixodes ricinus* (IR) and *Ixodes hexagonus* (IH) collected from 54 hedgehogs in the Campine region, Belgium (mean ± standard error, SE)

Of the 54 hedgehogs investigated, 24 were adults and 28 were juveniles. For two hedgehogs, age was not determined. Both *I. hexagonus* and *I. ricinus* ticks of all life stages were found on the hedgehogs. The number of ticks per hedgehog ranged from one to 167. Most hedgehogs in our study carried only few ticks, while only few individuals harboured most the ticks. Six of the 54 hedgehogs carried more than half of all ticks (624/1205) and only 15 hedgehogs carried 25 or more ticks. Tick burden did not significantly differ between hedgehog age classes (*χ* ^*2*^ = 0.001, *df* = 1, *P* = 0.97). In total, we collected 1205 ticks and found significantly more *I. hexagonus* (*n* = 1132) than *I. ricinus* (*n* = 73) (*χ* ^*2*^ = 29.126, *df* = 1, *P* = 0.0001) The most common life stage of *I. hexagonus* retrieved from the hedgehogs were nymphs (*n* = 586) (*χ* ^*2*^ = 4.2656, *df* = 1, *P* = 0.04). *I. ricinus*, all life stages were equally common (*χ* ^*2*^ = 3.1908, *df* = 1, *P* = 0.07, Fig. 1). Some hedgehogs were found to harbour both species of ticks (*n* = 10), but infestations with only one tick species were more common (*n* = 44,, *χ* ^*2*^ = 21.407, *df* = 1, *P* = 0.0001)

**Fig. 2:**
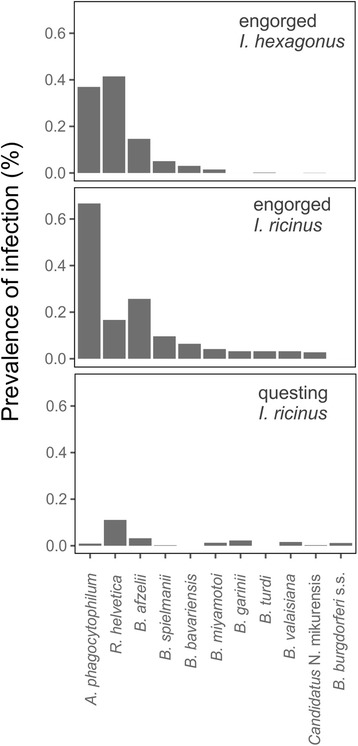
The prevalence of the distinct pathogens in *Ixodes ricinus* and *Ixodes hexagonus* ticks collected from hedgehogs (mean ± standard error, SE)

Of the 1205 collected ticks, two got lost during sample preparation, hence the molecular analyses were performed on 1203 ticks. A total of 859 (71.4%) ticks was infected with at least one of the tested pathogens. Of these infected ticks, 524 (61%) had a single infection, and 335 ticks (39%) were infected with more than one pathogen of another genus. The number and percentage of infected *I. ricinus* and *I. hexagonus* ticks per life stage are provided in Table 1. A more detailed overview, including the different *Borrelia burgdorferi* (*s.l*.) genospecies, is provided in Additional file 3. *Anaplasma phagocytophilum* and *R. helvetica* were the two most common pathogens and occurred in 466 ticks (38.7% of all analysed ticks or 54% of all infected ticks) coming from 34 hedgehogs and in 481 ticks (40% of all analysed ticks or 56% of all infected ticks) coming from 37 hedgehogs, respectively. An infection with *Borrelia burgdorferi* (*s.l*.) occurred in 297 ticks (24.7% of all analysed ticks or 34.6% of all infected ticks) from 28 hedgehogs. We could identify the *B. burgdorferi* (*s.l*.) genospecies in 129 (43.4%) of these infected ticks, of which *B. afzelii* (*n* = 80), *B. spielmanii* (*n* = 28) and *B. bavariensis* (*n* = 17) were the most common. *Borrelia turdi* occurred once in each tick species and *B. garinii* and *B. valaisiana* each in one *I. ricinus* tick. An infection with *B. miyamotoi* occurred in 20 ticks from five hedgehogs. Only three (one *I. hexagonus* and two *I. ricinus*) from two hedgehogs were infected with “*Ca.* Neoehrlichia mikurensis”. The pathogen prevalence per tick species is depicted in Fig. 2. All pathogens except for *R. helvetica* (*χ* ^*2*^ = 15.983, *df* = 1, *P* = 0.0001) were found more often co-existing with another pathogen in a tick, than as the single pathogen infecting the tick. *Ixodes ricinus* seems to be more likely infected with at least one pathogen (59/72, 81.9%) than *I. hexagonus* (800/1131, 70.7%) but the difference between the two tick species was only marginally significant (*χ* ^*2*^ = 3.6355, *df* = 1, *P* = 0.06). More specifically, the infection prevalence of *A. phagocytophilum*, “*Ca.* Neoehrlichia mikurensis”, *B. afzelii*, *B. garinii*, *B. valaisiana* and *B. turdi* was highest in *I. ricinus* while infection with *R. helvetica* was highest in *I. hexagonus* (*χ* ^*2*^ = 16.333, *df* = 1, *P* = 0.0001). For the infection prevalence of *B. miyamotoi*, *B. spielmanii* and *B. bavariensis*, no difference between the tick species could be observed. There was no difference in infection prevalence between adult and juvenile hedgehogs for any of the detected pathogens (*P* > 0.05) (Table 1).

**Table 1:** The number (#) of *Ixodes ricinus* and *Ixodes hexagonus* ticks infected with a certain pathogen, for all life stages together or for larvae (L), nymphs (N) or adults (A) separately, and the percentage (%) of infected ticks of the two species on all analyzed ticks from that species per life stage

			*B. burgdorferi* (*s.l*.)	*B. miyamotoi*	*R. helvetica*	*A. phagocytophilum*	“*Ca.* N. mikurensis”
*I. hexagonus*	L	No.	3	2	10	2	0
		%	6.3	4.2	20.8	4.2	0
	N	No.	166	8	192	279	1.0
		%	28.4	1.4	32.8	47.7	0.2
	A	No.	91	7	267	137	0
		%	18.3	1.4	53.6	27.5	0
	All	No.	260	17	469	418	1
		%	23.0	1.5	41.5	37.0	0.09
*I. ricinus*	L	No.	1	0	0	3	0
		%	14.3	0	0	42.9	0
	N	No.	20	0	3	23	2
		%	71.4	0	10.7	82.1	7.1
	A	No.	16	3	9	22	0
		%	43.2	8.1	24.3	59.5	0
	All	No.	37	3	12	48	2
		%	51.4	4.2	16.7	66.7	2.8

Co-infections of other pathogens with *B. burgdorferi* (*s.l*.) were investigated. For *I. ricinus*, 37 of the 59 infected ticks (62.7%) carried two (*n* = 31) or three (*n* = 6) pathogens. The most common co-infection in *I. ricinus* (24/37) was with *B. burgdorferi* (*s.l*.) and *A. phagocytophilum*. Of the 800 infected *I. hexagonus* ticks, 298 (37.3%) had a co-infection composed of two (*n* = 232) or three (*n* = 65) pathogens. Co-infections of *A. phagocytophilum* and *R. helvetica* (102/298), *A. phagocytophilum* and *B. burgdorferi* (*s.l*.) (86/298) and *A. phagocytophilum*, *R. helvetica* and *B. burgdorferi* (*s.l*.) (64/298) occurred most often. One *I. hexagonus* tick was infected with four pathogens: *A. phagocytophilum*, *R. helvetica*, *B. burgdorferi* (*s.l*.) and *B. miyamotoi*.

*Ixodes ricinus* larvae and nymphs from hedgehogs were infected more often (28/35) than questing *I. ricinus* nymphs and adults (367/1874) (*χ* ^*2*^ = 72.786, *df* = 1, *P* = 0.0001). We could not detect any difference in prevalence of *R. helvetica*, *B. miyamotoi*, *B. garinii* and *B. valaisiana*. For *A. phagocytophilum* (*χ* ^*2*^ = 807.24, *df* = 1, *P* = 0.0001), “*Ca.* Neoehrlichia mikurensis” (*χ* ^*2*^ = 14.989, *df* = 1, *P* = 0.0001), *B. afzelii* (*χ* ^*2*^ = 82.545, *df* = 1, *P* = 0.0001), *B. spielmanii* (*χ* ^*2*^ = 44.81, *df* = 1, *P* = 0.0001), *B. bavariensis* (*χ* ^*2*^ = 110.89, *df* = 1, *P* = 0.0001) and *B. turdi* (*χ* ^*2*^ = 110.89, *df* = 1, *P* = 0.0001), infection prevalence was significantly higher in the engorged ticks from the hedgehogs.

Afterwards we repeated these analyses for *I. hexagonus* collected from hedgehogs and compared the larvae and nymphs of this tick species with the questing *I. ricinus* nymphs and adults collected from the vegetation. This enables us to interpret more comprehensively the reservoir role of the hedgehog for the different pathogens, and the vector competence of *I. hexagonus*. We observed that *B. garinii* and *B. valaisiana* were more prevalent in the questing *I. ricinus* ticks. No significant difference in infection prevalence between questing or engorged ticks could be detected for *B. turdi*, *B. miyamotoi* and “*Ca.* Neoehrlichia mikurensis”. The prevalence of all other pathogens, including *R. helvetica,* is higher in the engorged than the questing ticks. Furthermore, when comparing just the ticks collected from hedgehogs that carried 25 or more ticks, we obtained the same outcome.

**Fig. 3:**
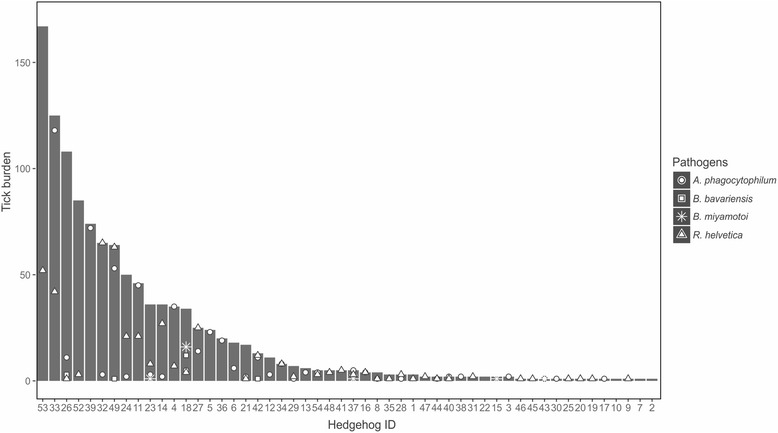
The tick burden per hedgehog with the number of ticks per hedgehog harbouring infection with *Anaplasma phagocytophilum*, *Rickettsia helvetica*, *Borrelia bavariensis* and *Borrelia miyamotoi*

For *A. phagocytophilum*, *R. helvetica*, *B. bavariensis* and *B. miyamotoi*, the distribution of the infections was clustered in some hedgehogs, with most hedgehogs harbouring no, or only few infected ticks, while only few hedgehogs were responsible for most of the infected ticks. This is visualized in Fig. 3. Twelve of the 17 ticks infected with *Borrelia bavariensis* and 16 of the 20 ticks infected with *B. miyamotoi* came from one individual hedgehog (hedgehog #18). Hedgehog #33 harboured a total of 125 ticks of which 118 were infected with *A. phagocytophilum* (25.3% of all *A. phagocytophilum* infections). Still, there are hedgehogs that harbour many ticks, while no or few or these ticks are infected with one of these pathogens (Fig. 3).

Of the *A. phagocytophilum* positive ticks, 43 were sequenced of which 33 *I. hexagonus* and 10 *I. ricinus* from 18 different individual hedgehogs. All the *groEL* sequences of the *A. phagocytophilum* isolates clustered with the zoonotic ecotype, ecotype I (not shown [44]).

## Discussion

Our results confirm that hedgehogs are a host of all three stages of *I. hexagonus* and *I. ricinus*. Still, more *I. hexagonus* were found feeding on hedgehogs than *I. ricinus* ticks (Fig. 1). The aggregation of ticks on hedgehogs varied vastly between the individual hedgehogs, as only a few hedgehogs were recorded to carry most of the ticks (Fig. 3). This means that just a few hedgehogs contribute to tick maintenance, similar to what is seen on rodents [47]. This seems to be especially the case for *I. hexagonus*, and to a lesser extend for *I. ricinus*, since the burdens of *I. ricinus* on hedgehogs appears to be relatively low. Moreover, it is less likely that hedgehogs can maintain the *I. ricinus* life-cycle as the sole host species because, even though it can feed all life stages of this generalist tick species, hedgehog densities in forested areas, the preferred habitat of *I. ricinus*, are too low [30, 31]. Namely, if all *I. ricinus* stages should rely only on the hedgehog to feed on, many ticks would starve and perish since the number of encounters with this host would be low. We believe, rather, that a host community without large mammals but composed only of small or medium sized hosts such as rodents, birds and hedgehogs (like in (sub) urban area’s and parks), can already be sufficient to complete the life-cycle of *I. ricinus*. This because, as we show, large mammals are not the only hosts adult *I. ricinus* ticks feed on. More research is needed, however, to elucidate the role of hedgehogs in the life-cycle of this generalist tick species.

Since 71.4% of the ticks retrieved from hedgehogs were infected by at least one pathogen, hedgehogs can be considered as amplifying hosts and epidemiologically important wildlife species. Moreover, 39% of all infected ticks carried more than one pathogen of another genus. High prevalence of tick-borne pathogens *B. bavariensis*, *B. spielmanii*, *B. afzelii*, *A. phagocytophilum* and *R. helvetica* in engorged *I. hexagonus* and *I. ricinus* ticks obtained from *E. europaeus*, indicates that hedgehogs contribute to pathogen maintenance in natural cycles in urban and suburban areas. For *B. bavariensis*, *B. spielmanii*, *B. afzelii*, *A. phagocytophilum* and *R. helvetica*, the infection prevalence was higher in the engorged ticks of both species, in comparison to the infection rates in questing ticks from the same region (Fig. 2). This indicates that the hedgehog is a possible reservoir host of these pathogens and contributes to their enzootic cycle. On the other hand, “*Ca.* Neoehrlichia mikurensis” infection rate was not significantly higher in questing *I. ricinus* ticks than in engorged hedgehog ticks, indicating that hedgehogs do not play a main role in the maintenance of the enzootic cycle of this pathogen.

Engorged *I. ricinus* ticks tend to be more infected with any pathogen in comparison to engorged *I. hexagonus*, except for *R. helvetica* which was significantly more prevalent in *I. hexagonus* ticks. Perhaps this observation can be subscribed to transmission pathway of *R. helvetica*, which occurs transovarially as well as transstadially. Therefore, ticks in nature are usually thought to be the main reservoir and vectors of *R. helvetica* [48]. However, since transovarial transmission rates are less than 100%, vertebrate hosts like the hedgehog can act as an amplifier of this pathogen, playing a vital role in transmission cycles. The pathogens that are present in engorged *I. ricinus* ticks can originate from a previous blood meal from another host species, while the pathogens *I. hexagonus* carries are most probably coming from the hedgehog, since hedgehogs are their preferred host species. This way infection prevalence in engorged *I. ricinus* can be higher than engorged *I. hexagonus*, when they fed in a previous stage on a host species that functions as an efficient reservoir species for some of the investigated pathogens, such as small rodents or birds.

Remarkably, the infection of some pathogens such as *B. bavariensis*, *B. miyamotoi*, *R. helvetica* and *A. phagocytophilum* seem to be clustered per individual hedgehog, meaning that only a few hedgehogs contribute to the gross of the infected ticks. *Borrelia miyamotoi* is known to give short-term systemic infection in rodents, therefore making rodents excellent but transitory amplifying hosts of this bacterium [49]. Vertebrates other than rodents may also become infected: *B. miyamotoi* DNA was also found in the tissue of a European greenfinch and a great tit [50]. The clustering of infected fed ticks on only one hedgehog in this study indicate that *B. miyamotoi* might result in a short-term systemic infection in hedgehogs as well. The role of these animals in the transmission cycle is not clear; they could be transitory hosts. Another possible explanation for the fact that many ticks were infected with the same pathogen on the same hedgehog could be co-feeding transmission [51, 52]. With this route of transmission, no systemic infection of the vertebrate host is necessary. The host is only a transient bridge, bringing together infected and uninfected ticks in both space and time, thereby facilitating pathogen exchange. The host does not necessarily have to be infected himself [51, 52]. The bird associated borreliae, *B. garinii* and *B. valaisiana*, were each detected in one *I. ricinus* adult tick, and *B. turdi* occurred in one *I. hexagonus* female and one *I. ricinus* nymph. We can thus confirm the indication that hedgehogs are no reservoir hosts for the bird associated, only for the rodent-associated, *B. burgdorferi* (*s.l*.) genospecies [15].

Hedgehogs and their host-specific parasite *I. hexagonus* seem to play a role in maintaining some pathogens, like *B. bavariensis*, *B. spielmanii*, and *A. phagocytophilum* in cryptic cycles. The generalist feeding behaviour of *I. ricinus* and the low prevalence of these pathogens in questing *I. ricinus* suggest that they do not play a main role in the maintenance of the enzootic cycle of these pathogens. However, when feeding on hedgehogs *I. ricinus* may still be infected by *I. hexagonus*-associated pathogens and transmit them to humans. *Borrelia bavariensis* can cause neurological disease in humans [7], and *B. spielmanii* has been linked to EM in humans. Both pathogens have already been linked to hedgehogs [15, 17]. Moreover, co-infection of *R. helvetica* and *B. burgdorferi* (*s.l*.) has been shown in neuroborreliosis patients [53]. Also, co-infections are thought to affect the severity of disease and influence clinical outcomes in some cases [54]. Since hedgehogs seem to be large contributors to co-infection rates in ticks, this poses an increased health risk. The variant of *A. phagocytophilum* detected in these samples were all linked to human cases of anaplasmosis (ecotype I) [44].

## Conclusions

From these findings, we conclude that hedgehogs are important components in the enzootic cycle of a diverse set of human pathogens, thereby contributing to the maintenance of various tick-borne diseases in (sub) urban areas. Humans are likely to encounter ticks infected with one or several of these pathogens while gardening or recreating in parks [55]. This poses a potential human health risk. Most hedgehogs, however, carry only few ticks and hedgehog densities are relatively low, thus hedgehogs will probably infect only few ticks with a certain pathogen. Further research is necessary to elucidate the interaction between hedgehog densities, tick burden and tick infection prevalence and to assess the precise impact of hedgehogs on the enzootic cycle of the various tick borne human pathogens, and the associated human health risk.

## Additional files

Additional file 1: IGS sequences obtained in this study. (XLSX 13 kb)
Additional file 2:
*groEL* sequences obtained in this study. (XLSX 10 kb)
Additional file 3: The number of *Ixodes ricinus* and *Ixodes hexagonus* ticks infected with a certain pathogen, for all life stages together or for larvae (L), nymphs (N) or adults (A) separately. (CSV 719 bytes)
